# Connexin 32 dysfunction promotes ethanol-related hepatocarcinogenesis via activation of Dusp1-Erk axis

**DOI:** 10.18632/oncotarget.6511

**Published:** 2015-12-09

**Authors:** Hiroyuki Kato, Aya Naiki-Ito, Taku Naiki, Shugo Suzuki, Yoriko Yamashita, Shinya Sato, Hiroyuki Sagawa, Akihisa Kato, Toshiya Kuno, Satoru Takahashi

**Affiliations:** ^1^ Department of Experimental Pathology and Tumor Biology, Nagoya City University Graduate School of Medical Sciences, Nagoya, Japan; ^2^ Department of Gastroenterological Surgery, Nagoya City University Graduate School of Medical Sciences, Nagoya, Japan; ^3^ Department of Gastroenterology and Metabolism, Nagoya City University Graduate School of Medical Sciences, Nagoya, Japan

**Keywords:** connexin 32, alcohol, hepatocarcinogenesis, Erk, Dusp1

## Abstract

There is abundant epidemiological evidence that heavy alcohol intake contributes to hepatocellular carcinoma (HCC) development. Previous reports indicated that connexin 32 (Cx32), which is a major hepatocyte gap junction protein, is down-regulated in chronic liver disease and has a protective role in hepatocarcinogenesis. However, functions of Cx32 in alcohol-related hepatocarcinogenesis have not been clarified. To evaluate them, 9-week-old Cx32 dominant negative transgenic (Tg) rats and their wild-type (Wt) littermates were given 1 % or 5 % ethanol (EtOH) or water *ad libitum*, for 16 weeks after an intraperitoneal injection of diethylnitrosamine (200 mg/kg). EtOH significantly increased the incidence and multiplicity of HCC and total tumors in a dose-dependent manner in Tg rats, but not in Wt rats. Although the number and area of glutathione S-transferase placental form (GST-P) positive foci were not significantly different between the groups, EtOH increased the Ki-67 labeling indices in GST-P positive foci only in Tg rats. EtOH up-regulated phosphorylated Erk1/2 with decrease of the Erk1/2 inhibitor, dual specificity protein phosphatase 1 (Dusp1) in whole livers of Tg and Wt rats. Immunofluorescence staining and quantitative RT-PCR revealed that EtOH significantly increased nucleolar localization of phosphorylated Erk1/2 and contrastingly reduced Dusp1 protein and mRNA expression in GST-P positive foci and HCC of Tg rats as compared to those of Wt rats. These findings suggest that Cx32 dysfunction like in chronic liver disease promoted EtOH-associated hepatocarcinogenesis through dysregulation of Erk-Dusp1 signaling.

## INTRODUCTION

Hepatocellular carcinoma (HCC) is the second most common cause of cancer-related mortality. An estimated 782,500 new liver cancer cases and 745,500 deaths occurred in 2012 worldwide [1]. Alcohol is one of the most important risk factor for the cause of HCC especially in developed countries, and the incidence of alcohol-related HCC has recently tended to increase in Japan [2]. In the recent monograph published by the International Agency for Research on Cancer (IARC), there is ‘sufficient evidence’ that demonstrate carcinogenicity of alcoholic beverage in humans (classified as Group 1), and the report concluded that the occurrence of primary liver cancer is casually related to alcohol intake [3]. Excessive alcohol consumption of >40 to 60 g/day for more than 5 years is a well-known factor that increases the risk of HCC by nearly 5-folds [4].

Several animal experiments have revealed that ethanol (EtOH) treatment promotes chemically-induced hepatocarcinogenesis [5–10], however, conflicting results regarding the mode of action of EtOH in hepatocarcinogenesis have also been reported [11]. Lifelong exposure to 3% EtOH did not induce HCC in rodents [12] whereas 10% EtOH ingestion for 18 months induced HCC development [13]. These results suggest that EtOH is an established carcinogen, which is consistent with the IARC evaluation of EtOH. However, the detailed molecular mechanisms by which EtOH contributes to hepatocarcinogenesis have not been fully elucidated to date.

Gap junctions formed by connexin hemichannnels exchange small molecules (<1kDa) between adjacent cells and play important roles in the maintenance of tissue homeostasis, the control of cell growth and differentiation [14, 15]. Hepatocytes express two connexin proteins, Cx32 which is the major protein and broadly expressed, and Cx26 which is localized at the periportal zone [16–18]. The expression of Cx32 was down-regulated in preneoplastic and HCC lesions in rats [19], and gradually decreased during progression of chronic liver disease including viral hepatitis, cirrhosis and HCC in humans [20, 21]. Reduction of Cx32 expression and gap junctional intercellular communication (GJIC) capacity also occurred in an age-dependent manner in rat [22]. We previously established transgenic rats, which carried a dominant negative mutant of Cx32 under the control of the albumin promoter (Tg) and harbored broadly disturbed membrane localization of endogenous Cx32 and Cx26 proteins and decreased GJIC as measured by gap junction assay. Tg rats are characterized as having higher susceptibility to DEN-induced hepatocarcinogenesis as compared with wild-type (Wt) littermate rats [23, 24]. These findings suggest that Cx32 has a protective role in hepatocarcinogenesis in rodents and humans. However, the relationship between Cx32 and EtOH-related hepatotoxicity including hepatocarcinogenesis has not been clarified.

In the present study, we examined the role of Cx32 in alcohol-related hepatocarcinogenesis using a Tg rat model to investigate the molecular mechanisms in preneoplastic and neoplastic lesions. Further, to assess the genes responsible for alcohol-related hepatocarcinogenesis, cDNA microarray analysis was performed.

## RESULTS

### EtOH does not induce liver injury in rats of both genotypes

**Figure 1:**
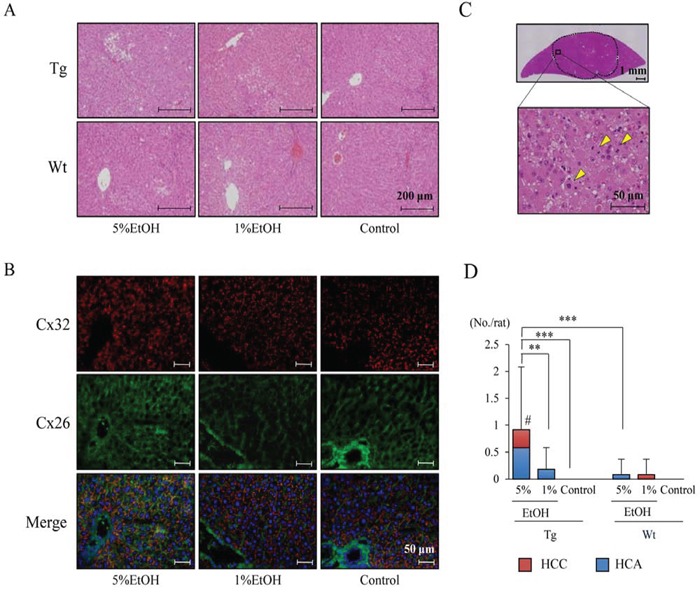
EtOH intake after DEN injection enhances hepatocarcinogenesis in Tg rats **A.** H&E staining of non-tumor region of the liver in each group. **B.** Immunofluorescence staining for Cx32 (red) and Cx26 (green) in livers of Wt rats. **C.** H&E staining of HCC in Tg rats given 5% EtOH. Mitotic cells are indicated by arrowheads. **D.** Multiplicity of HCC, HCA and total tumors. Data are presented as mean ± SD, *n* = 11–12 per group. **, *** *P* < 0.01 and 0.001, respectively. # *P* < 0.05 indicates statistical significance in HCC as compared to Tg-Control and Wt-5%EtOH.

EtOH drinking did not affect body weight in rats of both genotypes, and there was no significant difference in final body, liver and kidney weights among the groups. Average EtOH consumption was not significantly different between Tg and Wt rats (Table 1). In addition, EtOH did not affect serum levels of hepatic enzymes, lipids and albumin (Supplementary Table 1). Histological examination revealed that slight fat deposition was observed in the livers of Tg and Wt rats with EtOH treatment, however, there was no significant difference between the different genotypes (Figure 1A). EtOH induced expression of cytochrome P450 2E1 (Cyp2e1), which is one of the metabolic enzymes for EtOH, in the centrilobular region in a dose-dependent manner in both Tg and Wt rats, and protein expression of Cyp2e1 in this area was higher in Tg rats than that in Wt rats (Supplementary Figure 1A and 1B). The expression of Cx32 and Cx26, which are hepatocyte gap junction protein, were diffusely decreased in Tg rats as compared to those in Wt rats (Supplementary Figure 2A), and the expression of Cx32 and Cx26 on the cell membrane in Wt rats were not altered by EtOH intake (Figure 1B). The dye loading assay also revealed that there was no significant difference in the capacity of gap junction between each group in Wt rats (Supplementary Figure 2B and 2C).

**Table 1:** Final body, liver and kidney weights, and average EtOH intake in Tg and Wt rats

		No of rats		Alcohol intake	Liver		Kidney	
			Body(g)	(g/kg/day)	Absolute(g)	Relative(%)	Absolute(g)	Relative(%)
Tg	5%EtOH	12	491.7 ± 18.7	2.51 ± 0.45	13.7 ± 0.9	2.8 ± 0.1	1.2 ± 0.1	0.25 ± 0.02
	1%EtOH	11	509.6 ± 27.1	0.59 ± 0.07	14.6 ± 1.3	2.9 ± 0.2	1.2 ± 0.1	0.24 ± 0.01
	Control	12	522.4 ± 23.7	-	15.0 ± 1.1	2.9 ± 0.2	1.2 ± 0.1	0.24 ± 0.01
Wt	5%EtOH	12	507.2 ± 32.9	2.58 ± 0.30	14.0 ± 1.2	2.8 ± 0.2	1.2 ± 0.1	0.24 ± 0.02
	1%EtOH	12	535.4 ± 46.6	0.59 ± 0.07	15.1 ± 1.9	2.8 ± 0.2	1.3 ± 0.1	0.23 ± 0.01
	Control	12	542.3 ± 45.5	-	15.4 ± 1.4	2.8 ± 0.1	1.3 ± 0.1	0.23 ± 0.01

### EtOH promotes hepatocarcinogenesis in Tg rats

Induction of liver tumors was observed in both Tg and Wt rats that received EtOH. The liver tumors, which lost normal lobular architecture with solid cell growth and high mitotic counts in histology, were defined as HCC (Figure 1C). Most of the HCCs were found in the Tg-5%EtOH group. The incidence and multiplicity of HCC and/or HCA are shown in Table 2 and Figure 1D. The incidence of HCC and total tumors (HCA+HCA) was significantly increased by EtOH intake in a dose-dependent manner in Tg rats. On the other hand, induction of tumors was also observed in Wt rats that ingested EtOH, however, there was no significant correlation between tumor incidence and dosage of EtOH. Consequently, the incidence of HCC and total tumors was significantly higher in Tg rats as compared to that in Wt rats (Table 2). The multiplicity of HCC and total tumors was also significantly increased by EtOH intake only in Tg rats (Figure 1D).

**Table 2:** Incidence of hepatocellular carcinomas and adenomas in Tg and Wt rats

		Incidence of tumors		
		HCC	HCA	HCC + HCA
Tg	5%EtOH	3 (25%)* #	3 (25%)*	6 (50%)*** ##
	1%EtOH	0	2 (18%)	2 (18%)
	Control	0	0	0
Wt	5%EtOH	0	1 (8%)	1 (8%)
	1%EtOH	1 (8%)	0	1 (8%)
	Control	0	0	0

Data are presented as mean ± SD. *, *** *P* < 0.05, 0.001 indicates statistical significance as compared to Tg-Control group. #, ## *P* < 0.05, 0.01 indicates statistical significance as compared to Wt-5%EtOH.

### EtOH enhances cell proliferation in preneoplastic lesions in Tg rats

**Figure 2:**
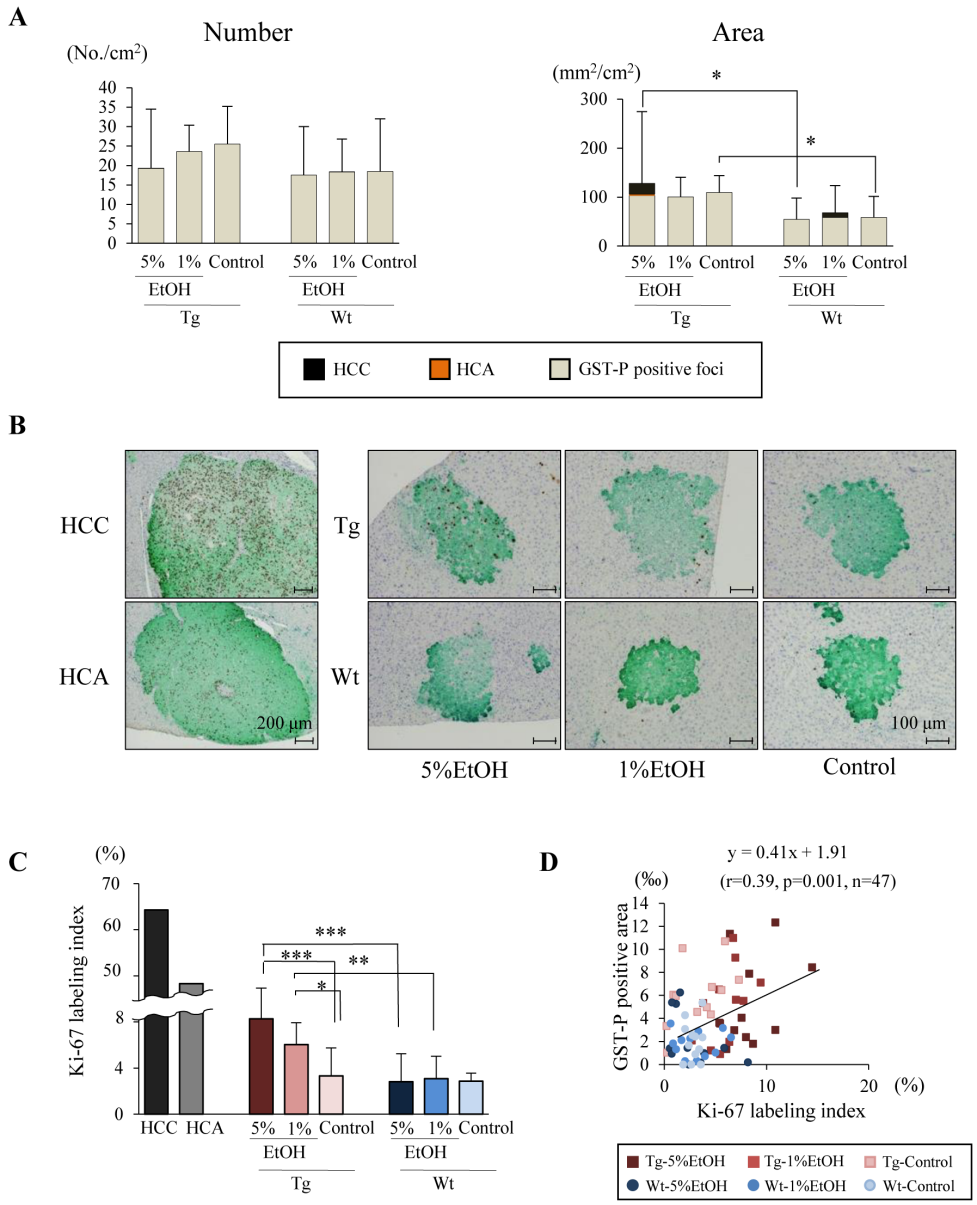
EtOH intake promotes cell proliferation in GST-P positive foci of Tg rats **A.** Number and area of GST-P positive lesions including foci, HCA and HCC. **B.** Double immunohistochemical staining for Ki-67 (brown) and GST-P (green). **C.** Ki-67 labeling indices in HCC, HCA and GST-P positive foci of each group. **D.** Calculation of the regression line based on the relationship between Ki-67 labeling index and GST-P positive area by Pearson's correlation (*r* = 0.39, *p* = 0.001, *n* = 47). Data are presented as mean ± SD, *n* = 11–12 per group. *, **, ***: *P* < 0.05, 0.01, and 0.001 respectively.

Total glutathione S-transferase placental form (GST-P) positive areas including GST-P positive foci (diameter > 200 μm) as preneoplastic lesions, HCA and HCC were increased in Tg rats as compared to those in Wt rats regardless of EtOH intake, and tended to increase with EtOH intake only in Tg rats. However, EtOH intake did not affect the number and area of GST-P positive foci among each group in both Tg and Wt rats (Figure 2A). According to these results, we hypothesized that EtOH promotes hepatocarcinogenesis by elevation of proliferation in hepatic preneoplastic foci. To verify the hypothesis, we measured Ki-67 labeling index in GST-P positive foci of each group. The index were significantly increased by EtOH in a dose-dependent manner in Tg rats, and correlated with tumor incidence and multiplicity. In contrast, EtOH did not affect the Ki-67 labeling indices in GST-P positive foci of Wt rats, indicating that EtOH increased proliferation activity only in Tg rats (Figure 2B and 2C). The Ki-67 labeling index was substantially higher in HCC than in GST-P positive foci, and correlation between GST-P positive area and Ki-67 labeling index was observed (Pearson correlation coefficient, r=0.39, p=0.0001, n=71) (Figure 2D). These results suggest that EtOH may contribute to the promotion phase during hepatocarcinogenesis.

### EtOH activates Erk signaling pathway in Tg rats

**Figure 3:**
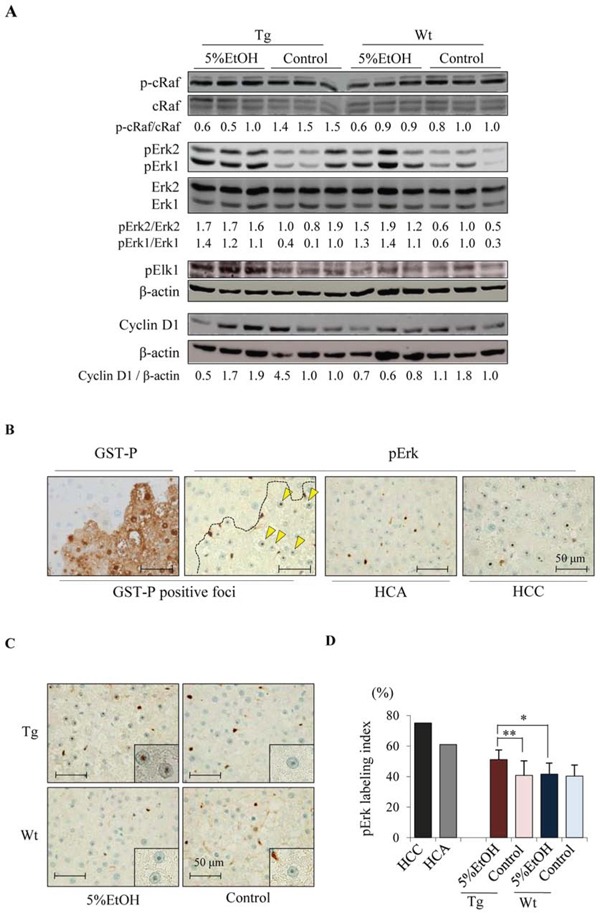
EtOH intake activates Erk signaling pathway in Tg rats **A.** Western blotting analysis for components of the Erk pathway including p-c-Raf (Ser338), c-Raf, Erk1/2, pErk1/2 (Thr202/Tyr204), pElk1 (Ser383), and cyclin D1. **B.** GST-P and pErk in liver of Tg rat given EtOH. Nucleolar pErk is indicated by arrowheads. **C.** Immunohistochemical staining for pErk in GST-P positive foci of each group. **D.** The percentage of nucleolar pErk positive hepatocytes in HCC, HCA of Tg-5%EtOH group and GST-P positive foci of each group. Date are presented as mean ± SD, *n* = 11–12 per group. *,**: *P* < 0.05 and 0.01 respectively.

To elucidate how EtOH promotes increased cell proliferation during hepatocarcinogenesis in Tg rats, we examined the expression of proteins in the mitogen-activated protein kinase (MAPK) pathway that play crucial roles in tumorigenesis. Western blot analyses revealed that Erk1/2 were activated in the liver of Tg and Wt rats given EtOH, although c-Raf was not affected. In addition, phosphorylated Elk1 (pElk1) and cyclin D1 expression were increased by EtOH intake in Tg rats, but not in Wt rats (Figure 3A). These results suggest that EtOH promotes cell proliferation in Tg rats through the Erk signaling pathway. Next, we analyzed the localization of activated Erk in HCC, HCA, GST-P positive foci and adjacent non-tumor tissue in the liver of each group. Immunohistochemical staining revealed that phosphorylated Erk 1/2 (pErk) was found in the nucleoli of neoplastic lesions as well as GST-P positive foci in each group although nucleolar pErk was not observed in normal hepatocyte (Figure 3B). The frequency of nucleoli-localized pErk in GST-P positive foci was significantly increased by EtOH intake only in Tg rats (Figure 3C and 3D). We also examined the expression of activated protein 1 (AP-1) transcription factors including c-Fos and c-Jun, which are downstream targets of Erk signaling and known to play pro-oncogenic roles in HCC [25]. Quantitative RT-PCR revealed that there was no significant difference in the mRNA expression of these two molecules between each group (Supplementary Figure 3A). Phosphorylated c-Jun (p-c-Jun) was increased in GST-P positive foci as compared to that in surrounding non-tumor tissues, however, EtOH did not affect p-c-Jun levels in GST-P positive foci in both Tg and Wt rats (Supplementary Figure 3B). These observations suggest that other molecules rather than c-Jun and c-Fos contribute to the promotion of cell proliferation by Erk during alcohol-related hepatocarcinogenesis.

### EtOH and dysfunction of Cx32 down-regulate Dusp1 expression in preneoplastic foci

**Figure 4:**
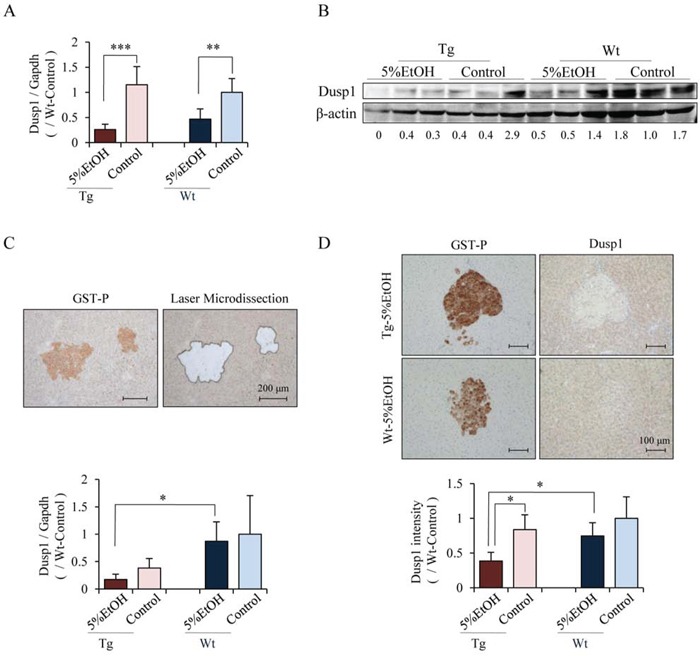
The mRNA and protein levels of Dusp1 were decreased by EtOH in Tg rats especially in GST-P positive foci Levels of Dusp1 mRNA **A.** and protein **B.** in whole liver tissues measured by quantitative RT-PCR and western blotting, respectively. Data of quantitative RT-PCR are presented as mean ± SD, *n* = 4 per groups. **, ***: *P* < 0.01 and 0.001 respectively. Representative image of laser microdissected tissue (upper) and Dusp1 mRNA level in GST-P positive foci as determined by quantitative RT-PCR (lower) **C.** Immunohistochemical staining for Dusp1 in GST-P positive foci of Tg and Wt rats that received 5%EtOH (upper) and the intensity score of Dusp1 in GST-P positive foci as compared to Wt-control rats (lower) **D.** Data are presented as mean ± SD, *n* = 5 per group, 10 foci/rat. *: *P* < 0.05.

**Figure 5:**
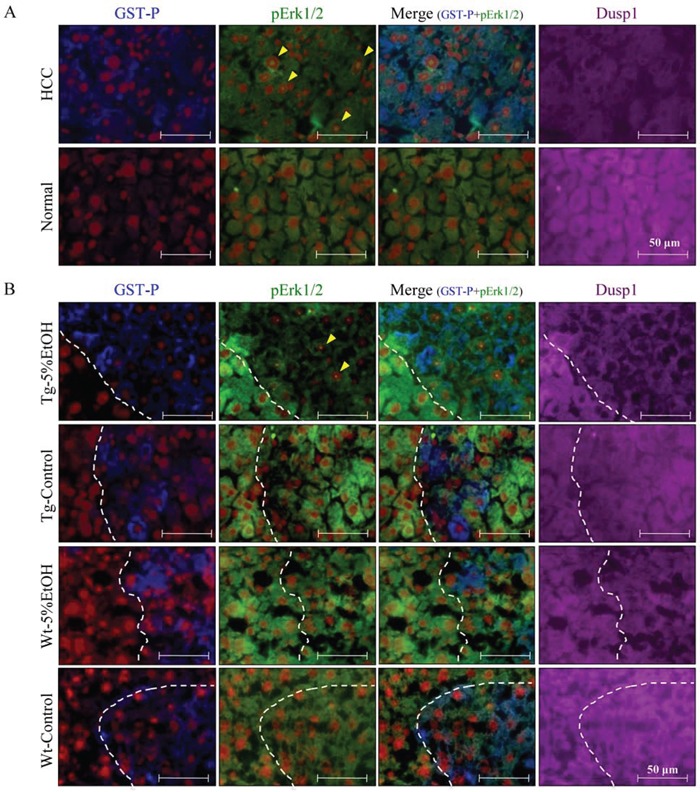
Localization of GST-P (blue), pErk (green), Dusp1 (purple), and nuclei (red) by triple immunofluorescence staining in HCC and normal tissue of the Tg-5%EtOH group **A.** and GST-P positive foci of each group **B.** Nucleolar pErk positive hepatocytes are indicated by arrowheads.

To further clarify the mechanisms of alcohol-related hepatocarcinogenesis, cDNA microarray analysis was performed using liver tissues of 5%EtOH and control groups of each genotype. Genes that were up-regulated or down-regulated more than 2-folds by EtOH treatment in Tg rats are listed in Table 3. Among these genes, we focused on Dusp1 and Dusp4, whose expression levels were decreased by EtOH in Tg rats, because some members of Dusp family are known to be inhibitors of MAPK signaling including the Erk pathway. Quantitative RT-PCR confirmed that Dusp1 mRNA expression was down-regulated by EtOH treatment in both genotypes, and immunoblot analyses demonstrated that Dusp1 protein expression tended to be decreased by EtOH in both genotypes, especially in Tg rats (Figure 4A and 4B). On the other hand, the mRNA expression of Dusp4, another member of the Dusp family, was too low and no significant difference was detected among the groups by quantitative RT-PCR (data not shown). Therefore, further analyses focused only on Dusp1 expression especially in GST-P positive foci, since EtOH increased nucleoli-located pErk in GST-P positive foci of Tg rats compared with that of Wt rats. The mRNA and protein levels of Dusp1 in GST-P positive foci were significantly decreased in Tg rats treated with EtOH as compared to those in treatment-matched Wt rats. EtOH intake also significantly decreased the Dusp1 protein expression as compared to the no treatment group of Tg rats (Figure 4C and 4D). To elucidate the relationship between localization of pErk and Dusp1 in HCC and GST-P positive foci, we performed triple immunofluorescence staining for pErk, Dusp1 and GST-P. Expression of Dusp1 was lower in HCC than in normal hepatocytes (Figure 5A). In addition, lower Dusp1 expression was observed in GST-P positive foci than that in normal hepatocytes in the Tg-5%EtOH group. Moreover, the Dusp1 expression level was inversely correlated with pErk expression in nucleoli in both HCC and GST-P positive foci of Tg rats that received EtOH (Figure 5B). These results suggest that EtOH treatment and Cx32 dysfunction decreased Dusp1 expression which led to Erk activation in GST-P positive foci. Therefore, the interaction of Erk and Dusp1 may be involved in the promotion of EtOH-related hepatocarcinogenesis in Tg rats.

**Table 3:** Up-regulated and down-regulated genes in the liver of EtOH-treated Tg rats

Reference ID	Symbol	Description	Ratio
Up-regulated genes		(Tg-5%EtOH/Tg-Control)	
NM_019156.2 NM_012879.2 NM_130752.1 NM_019130.1 NM_001004084.1 NM_001004253.1 NM_001033866.1	Vtn Slc2a2 Fgf21 Ins2 RT1-Bb Syap1 Surf2	Vitronectin Solute carrier family 2, facilitated glucose transporter member 2 Fibroblast growth factor 21 Insulin-2 precursor RT1 class II histocompatibility antigen, B-1 beta chain precursor Synapse associated protein 1 Surfeit 2	13.58 8.02 3.30 3.17 2.29 2.22 2.22
Down-regulated genes			
NM_024385.1 NM_022199.1 NM_022671.2 NM_012543.2 NM_053769.3 NM_053328.1 NM_058208.1 NM_153724.2 XM_227134.4 NM_001077640.1 XM_343472.3 NM_019138.1 NM_080886.1 NM_017136.2 NM_053445.2 NM_001034950.1 NM_001013072.1 NM_001039023.1 NM_001012111.1	Hhex Dusp4 Onecut1 Dbp Dusp1 Bhlhb2 Socs2 Dscr1 Ccrn4l Gadd45g Cish Cyp7b1 Sc4mol Sqle Fads1 Rup2 Sfxn2 Net1 Lpin1	Hematopoietically expressed homeobox Dual specificity protein phosphatase 4 Hepatocyte nuclear factor 6 D site-binding protein Dual specificity protein phosphatase 1 Class B basic helix-loop-helix protein 2 Suppressor of cytokine signaling 2 Calcipressin-1 Nocturnin (CCR4 protein homolog) (Fragment) Growth arrest and DNA-damage-inducible protein GADD45 gamma Cytokine-inducible SH2-containing protein Cytochrome P450 7B1 C-4 methylsterol oxidase Squalene monooxygenase Fatty acid desaturase 1 Urinary protein 2 Sideroflexin 2 Neuroepithelial cell transforming gene 1 Lipin 1	0.23 0.24 0.25 0.26 0.27 0.28 0.28 0.36 0.37 0.40 0.41 0.42 0.42 0.44 0.46 0.47 0.49 0.49 0.49

## DISCUSSION

In the present study, we clearly demonstrated the enhancing effects of EtOH on DEN-induced hepatocarcinogenesis via Cx32 dysfunction. These results are considered to reflect the strong association of alcohol intake with HCC development in patients with chronic hepatitis since Cx32 is down-regulated in hepatitis, cirrhosis and aging. EtOH intake in rats of the 5% EtOH group was approximately 2.5 g/kg/day which is equivalent to 150 g/day for a human who is 70 kg in weight. However, since the activity of the alcohol metabolizing enzyme, alcohol dehydrogenase, in rat liver has been reported to be 4-5 times higher than that in human liver [26], the equivalent ingested amount would be 30-37.5 g/day/70 kg person after adjusting for the difference in metabolizing enzyme activity. This is speculated to be the marginal level of EtOH intake for elevating the risk of alcohol-related HCC development in human. In fact, only a small number of tumors were observed in Wt rats in contrast to Tg rats that were susceptible to hepatocarcinogenesis.

One effect of EtOH in the whole livers of both genotypes was up-regulation of pErk protein expression, while pElk1 and cyclin D1 were increased by EtOH treatment only in Tg rats. Since a previous paper suggests that alcohol consumption increases the percentage of pErk-positive sinusoidal lining cells in rats [13], the labeling index in sinusoidal lining cells was measured in this study. The pErk-positive sinusoidal lining cells were significantly increased by EtOH treatment in both Tg and Wt rats, and there was no significant difference between genotypes (Supplementary Figure 4A), which was consistent with the results by western blot analysis. The Ki-67 labeling index in sinusoidal lining cells was also analyzed to investigate whether pErk induction affects proliferative activity in these cells, and we found that it was not altered among the groups (Supplementary Figure 4B). Concerning to the expression of pErk in GST-P positive foci, EtOH increased the percentage of nucleoli-localized pErk and Ki-67 positivity in hepatocytes of GST-P positive foci in Tg rats, and there effects were not observed in Wt rats. Elk1 is known to be activated by phosphorylated Erk, p38 Mapk or Sapk/Jnk [27], and only Erk was activated among these MAPK (Supplemantary Figure 5) in our study. Several reports have described the relationship of Cx32 with cell proliferation and cyclin D1 expression: (a) proteome analysis indicated that Cx32 indirectly interacted with Erk [28], (b) radiation-induced liver tumors in Cx32 knock-out mice had increased pErk staining compared with that in Wt mice [29], and (c) overexpression of Cx32 decreased cyclin D1 expression and caused G1 arrest in liver cancer cells [30]. These reports and our data in the present study suggest that nucleolar pErk may induce expression of pElk1 and cyclin D1, promote cell proliferation in GST-P positive foci and increase incidence of HCC only in Tg but not Wt rats although the detailed mechanisms and intermediary factors are not known.

Therefore, microarray analysis was performed to elucidate these factors, and we focused on Dusp1 as a candidate regulator of pErk via Cx32 dysfunction in EtOH-related hepatocarcinogenesis, because Dusp1 is known to inhibit MAPKs including Erk. Dusp1 is known to inhibit MAPKs including Erk, and its down-regulation was reported to be a valuable factor for poor prognosis in HCC patients [31]. It has been demonstrated that Dusp1 expression in the liver is associated with the difference in susceptibility of different strains of rats to hepatocarcinogenesis [32]. Recently, Lawan et al. demonstrated an increase in hepatic lipogenesis and inhibition of CREB-mediated glucogenesis in liver-specific Dusp1 knock-out mice, which indicate that Dusp1 also contributes to steatosis including ALD and nonalcoholic fatty liver disease (NAFLD) [33]. Therefore, Dusp1 may be a potential target of prevention of the development of EtOH-related HCC as well as ALD and NAFLD.

It has been reported that the expression levels of both Dusp1 and Cx32 are positively regulated by S-adenosylmethionine (SAM) in hepatocytes [34, 35]. Methionine adenosyltransferase 1 alpha (MAT1A), which encodes the SAM synthesizing enzyme, was demonstrated to be reduced in the patients with ALD, and MAT1A knock-out mice showed increased susceptibility to hepatocarcinogenesis leading to development of HCC [36]. SAM exerted preventive effects in chemically-induced rat hepatocarcinogenesis and in an orthotopic-inoculated HCC rat model [37, 38]. These findings suggest that the chemopreventive and therapeutic effects of SAM are through Dusp1-Erk and Cx32, and it is likely that the Cx32-Dusp1-Erk signaling pathway was deeply involved in the promotion of hepatocarcinogenesis by EtOH in our Tg rat model.

With regard to the molecular mechanisms by which EtOH contributes to hepatocarcinogenesis *in vivo* models, Mercer et al. demonstrated that the Wnt/β-catenin signal pathway was activated in EtOH-related DEN-induced hepatocarcinogenesis in mice [9]. They reported that multiplicities of both eosinophilic liver cell foci and hepatocellular adenoma were significantly increased by EtOH treatment whereas HCC was not increased. These observations suggested that the Wnt/β-catenin signal pathway was not associated with malignant transformation in EtOH-related hepatocarcinogenesis. In contrast to their findings, it is likely that the Cx32-Dusp1-Erk signaling axis is profoundly involved in the entire carcinogenic process throughout all of the progression steps; namely, Dusp-Erk signaling is activated even in preneoplastic lesion in our present study.

In conclusion, Cx32-Dusp1-Erk interaction may contribute to the tumor promoting activity of EtOH and subsequent development of hepatocarcinogenesis. The data in the present study provide evidence that the Cx32-Dusp1-Erk signaling pathway is a potential target for chemoprevention and alternative therapy in EtOH-related hepatocarcinogenesis. In addition, the Cx32 dominant negative transgenic rats used in this study may be a useful *in vivo* model to study alcohol-related hepatocarcinogenesis because HCC can be induced by EtOH in a short period of time.

## MATERIALS AND METHODS

### Animal experiment

The establishment, production and screening of Tg rats carrying the mutated Cx32 gene were as previously described in detail [23]. Male Tg rats were produced by mating heterozygous males with Wt Sprague-Dawley females (Japan SLC, Shizuoka, Japan). Rats were maintained in plastic cages on hardwood chips in an air-conditioned specific pathogen-free animal room at 22 ± 2°C and 50% humidity with 12h/12h light-dark cycle. All animal experiments were performed under protocols approved by the Institutional Animal Care and use Committee of Nagoya City School of Medical Sciences. All heterozygous male Tg and Wt littermate rats were administrated a single intraperitoneal injection of 200 mg/kg DEN (Tokyo Kasei Kogyo Co, Ltd., Tokyo, Japan) dissolved in saline at 9 weeks of age. Thereafter they received 1 % or 5 % EtOH (Wako Pure Chemical Industries, Ltd., Osaka, Japan) or water *ad libitum* for 16 weeks: Tg-5%EtOH, Tg rats drinking 5% EtOH (n=12); Tg-1%EtOH, Tg rats drinking 1% EtOH (n=12); Tg-Control, Tg rats drinking water (n=12); Wt-5%EtOH, Wt rats drinking 5% EtOH (n=12); Wt-1%EtOH, Wt rats drinking 1% EtOH (n=12); and Wt-Control, Wt rats drinking water (n=12). All rats were sacrificed at the sixteenth week following the initiation of treatment.

### Biochemical analysis

Blood was collected by puncture of the abdominal aorta in anesthetized rats and separated serum by centrifugation (3,000 rpm) was transferred into tubes. Plasma albumin, alkaline phosphatase, aspartate aminotransferase, alanine aminotransferase, gamma-glutamyl transpeptidase, lactate dehydrogenase, and total cholesterol were determined by The Tohkai Cytopathology Institute: Cancer Research and Prevention (Gifu, Japan).

### Histological analysis of the livers

The livers were immediately excised, weighed and cut into slices 3 to 4 mm thick. They were then fixed in 10% buffered formalin, embedded in paraffin and routinely processed for histological evaluation (3 μm thick). Sections were stained with hematoxylin and eosin (H&E), and were also used for immunohistochemistry using anti-Cyp2e1 (Enzo Biochem Inc., New York, NY), anti-GST-P (Medical & Biological Laboratories, Nagoya, Japan), anti-pErk (Thr202/Tyr204), anti-p-c-Jun (Ser73) (Cell Signaling Technology, Danvers, MA), anti-Ki-67 (Abcam plc, Cambridge, UK) antibodies and anti-Dusp1 (Santa Cruz Biotechnology Inc., Santa Cruz, CA) antibodies. Double immunostaining for Ki-67 and GST-P or Ki-67 and p-c-Jun were performed using a previous method with modifications [39]. The section was incubated with a primary antibody against Ki-67 and visualized with DAB, and then the primary antibody was inactivated by heat treatment (95°c) for 10 min in 10 mM citrate buffer (pH 6.0). Thereafter the section was incubated with a second antibody against GST-P or p-c-Jun and visualized with the Vina Green Chromogen Kit (Biocare Medical, LLC. Concord, CA). The average number and area of GST-P positive foci whose size was more than 200 μm in diameter, and the total area of the liver section were measured with an image analyzer (Keyence, Osaka, Japan) (n=11-12). The proportion of hepatocytes positive for Ki-67, pErk, and p-c-Jun in GST-P positive foci (n=11-12), and the population of sinusoid lining cells positive for Ki-67 and pErk (n=5) were measured by counting at least 1,000 cells. The intensity score of Dusp1 immunostainings was evaluated using an image analyzer and associated software (Keyence), and was represented as a value relative to the Wt-control group (n=5 and 10 foci/rat, respectively).

### Immunofluorescence staining for Cx32 and Cx26

The detailed methods for fluorescence immunohistochemistry employed in this study have been described previously [22]. Frozen sections were cut to 6 μm thickness and fixed in cold acetone and 10% buffered formalin. A polyclonal rabbit antibody against Cx32 (Thermo Fischer Scientific Inc., Waltham, MA) was used with biotin-conjugated anti-rabbit IgG and TRITC-labeled streptavidin (Thermo Fischer Scientific Inc.) to visualize the endogenous proteins using an image analyzer (Keyence). A monoclonal mouse antibody against Cx26 (Thermo Fischer Scientific Inc.) was used with biotin-conjugated anti-mouse IgG and FITC-labeled streptavidin (Thermo Fischer Scientific Inc.).

### Triple immunofluorescence immunohistochemistry for pErk, Dusp1 and GST-P

Immunofluorescence staining for pErk, Dusp1 and GST-P was performed according to a modified protocol [39]. A monoclonal rabbit antibody for pErk (Cell Signaling Technology) was used with biotin-conjugated anti-rabbit IgG and FITC-labeled streptavidin (Thermo Fischer Scientific Inc.) in an immunoreaction enhancer solution, Can Get Signal (TOYOBO, Osaka, Japan), and then incubated at 95°c for 10 min in 10 mM citrate buffer (pH 6.0). Thereafter a polyclonal rabbit antibody against GST-P was used with biotin-conjugated anti-rabbit IgG and Cy5-labeled streptavidin (Thermo Fischer Scientific Inc.), and finally the section was incubated with an Alexa Fluor 350 conjugated rabbit anti-DUSP1 polyclonal antibody (Bioss Inc., Boston, MA). The nuclei were stained with Propidium Iodide (Vector Laboratories, Inc., Burlingame, CA).

### Gap junction assay

This procedure was performed according to the method described in an earlier report [40]. Briefly, liver samples were obtained from Wt rats with each treatment (4 rats per group) and treated with Lucifer yellow (Sigma-Aldrich Corp., St. Louis, MO), a stain that can pass through the gap junction channel, and rhodamine-dextran (Sigma-Aldrich Corp.), which does not cross through the channel, to measure gap junction capability. Liver slices were cut to 5 mm-thick and 3 incisions of 1 mm depth were made, followed by the addition of a mixture of fluorescent dyes containing 0.05% Lucifer yellow and 0.05% rhodamine-dextran in PBS into the incisions. After 3 minutes, the slices were washed 3 times with PBS and frozen. Thereafter 7 μm thick frozen sections were made and spread of the dye was measured using an image analyzer (Keyence).

### Western blotting

Liver tissues were homogenized with T-PER Tissue Protein Extraction Reagent (Thermo Fischer Scientific Inc.) and a protease inhibitor cocktail (Roche Diagnostic, Mannheim, Germany) on ice. Protein concentrations were determined by the Bradford method using a protein assay kit (Bio-Rad laboratories, Hercules, CA). Samples of 50 μg were mixed with SDS sample buffer, heated at 100°C for 10 min and then subjected to SDS-PAGE. The proteins were separated in 12% acrylamide gels and then transferred onto Hybond-ECL membranes (GE Healthcare UK Ltd., Buckinghamshire, UK). The antibodies used were against phosphorylated c-Raf (Ser338) (p-c-Raf), Erk 1/2, pErk, p38Mapk, phosphorylated p38Mapk (Thr180/Tyr182) (p-p38Mapk), pElk1 (Ser383), Sek1/Mkk4, phosphorylated Sek1/Mkk4 (Ser80) (pSek1/Mkk4), Sapk/Jnk, phosphorylated Sapk/Jnk (Thr183/Tyr185) (pSapk/Jnk), cyclin D1, Dusp1 (Cell Signaling Technology), c-Raf (BD bioscience, Franklin Lakes, NJ), and Cyp2e1 (Enzo Biochem Inc.). Equal protein loading was ascertained by western blotting with a β-actin antibody (Sigma-Aldrich corp.).

### Quantitative RT-PCR

Total RNA was extracted using Isogen reagent (Nippon Gene Co. Ltd., Tokyo, Japan). One microgram of RNA was converted to cDNA with avian myeloblastosis virus reverse transcriptase (Takara, Otsu, Japan) in 20 μl reaction mixture. Aliquots of 2 μl of cDNA samples were subjected to quantitative PCR in a total volume of 25 μl using SYBR Premix ExTaqII (Takara) in a light cycler apparatus (Roche Diagnostic). Primers used for amplification of each mRNA were as follows:

Dusp1 forward, 5′-TGTAGCACCCCTCTCTACGA-3′; Dusp1 reverse, 5′-GACAATTGGCCGAGACGTTG-3′; c-Jun forward, 5′-TCATCCAGTCCAGCAATGGG-3′; c-Jun reverse, 5′-TATGCAGTTCAGCTAGGGCG-3′; c-Fos forward, 5′-ACCACGACCATGATGTTCTC-3′; c-Fos reverse, 5′-GACAGATCTGCACAAAAGTC-3′.

### Microarray analysis

Gene expression analysis was performed using a Rat Oligo chip 20k (Toray Industries, Tokyo, Japan) according to the manufacturer's instructions. Hepatic RNA expression of the 4 experimental groups (Tg-5%EtOH, Tg-Control, Wt-5%EtOH and Wt-Control) was compared.

### Laser microdissection

The detailed methods employed in this study have been described previously [41]. Seven-micron thick frozen sections (6-8 sections) were mounted onto slides with films (Carl Zeiss, Oberkochen, Germany) and fixed in acetone for 10 min. The sections were treated with a polyclonal rabbit antibody against GST-P with RNaseOut (Thermo Fischer Scientific Inc.) for 25 min subsequently exposed to secondary antibody using the DAKO ENVISION™ System (DAKO Co., Tokyo, Japan) with RNaseOut and 2.5 μM EDTA for 25 min at 4°C and visualized with DAB after incubation for 5 min at room temperature. After immunostaining of the frozen tissue, laser microdissection was performed using the PALM MicroBeam system (Carl Zeiss) and 30 – 40 GST-P positive foci were collected into tubes. Thereafter, we quantified the mRNA level by quantitative RT-PCR as described above.

### Statistical analysis

Differences in the quantitative data, expressed as mean ± SD, between groups were compared by one-way ANOVA and Dunnett's post-hoc test using Graph Pad Prism 5 (GraphPad Software, Inc., La Jolla, CA).

## SUPPLEMENTARY FIGURES AND TABLE


