# Supplementary material for: Connexin 32 dysfunction promotes ethanol-related hepatocarcinogenesis via activation of Dusp1-Erk axis
Source: Oncotarget. 2015 Dec 9;7(2):2009–21. doi: 10.18632/oncotarget.6511 (PMC4811513; doi:10.18632/oncotarget.6511)
Supplement: Supplementary file 1 [file oncotarget-07-2009-s001.pdf]

SUPPLEMENTARY FIGURES AND TABLE

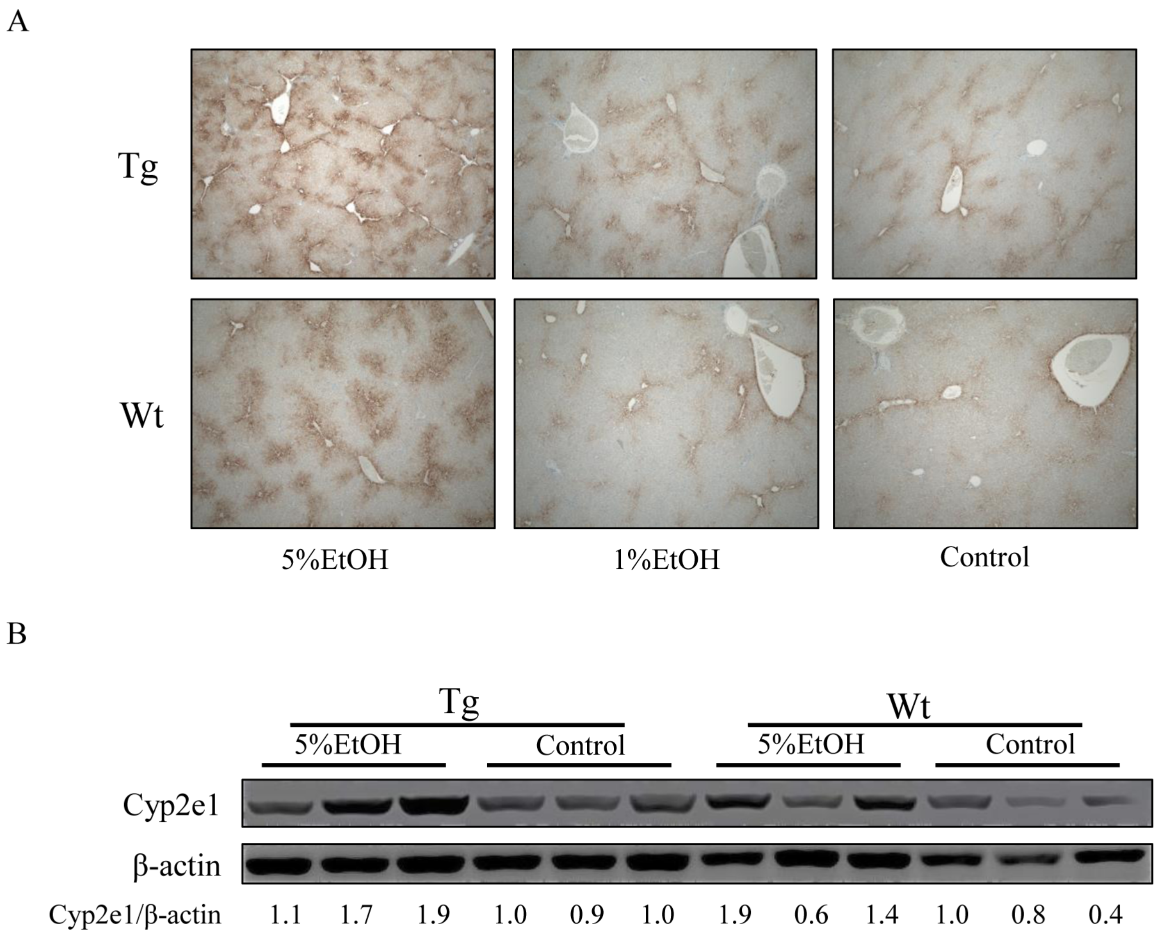

Supplementary Figure S1: EtOH intake induces Cyp2e1 expression in centrilobular region and higher expression in Tg rats as compared to Wt rats. Immunohistochemical staining **A.** and western blotting **B.** for Cyp2e1.

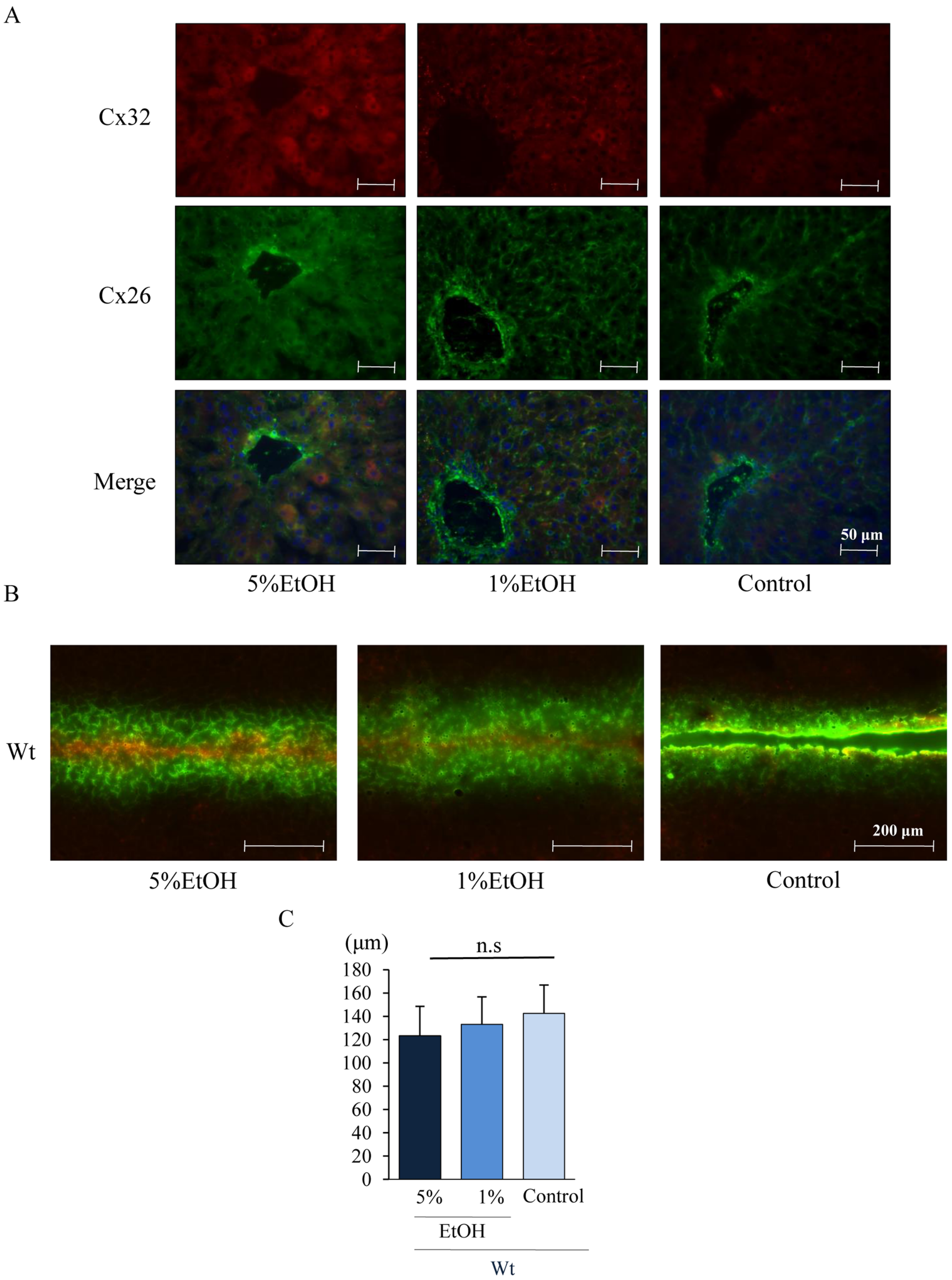

Supplementary Figure S2: GJIC capacity tended to decrease by EtOH intake but no significant difference was observed in Wt rats. A. Immunofluorescence staining for Cx32 (red) and Cx26 (green) in livers of Tg rats. **B.** Spread of Lucifer yellow through gap junction. Merged pictures of Lucifer yellow (green) and rhodamine-dextran (red), and **C.** distance of Lucifer yellow spread (μm) in Wt rats. Data are presented as mean ± SD, $n = 5$ per group.

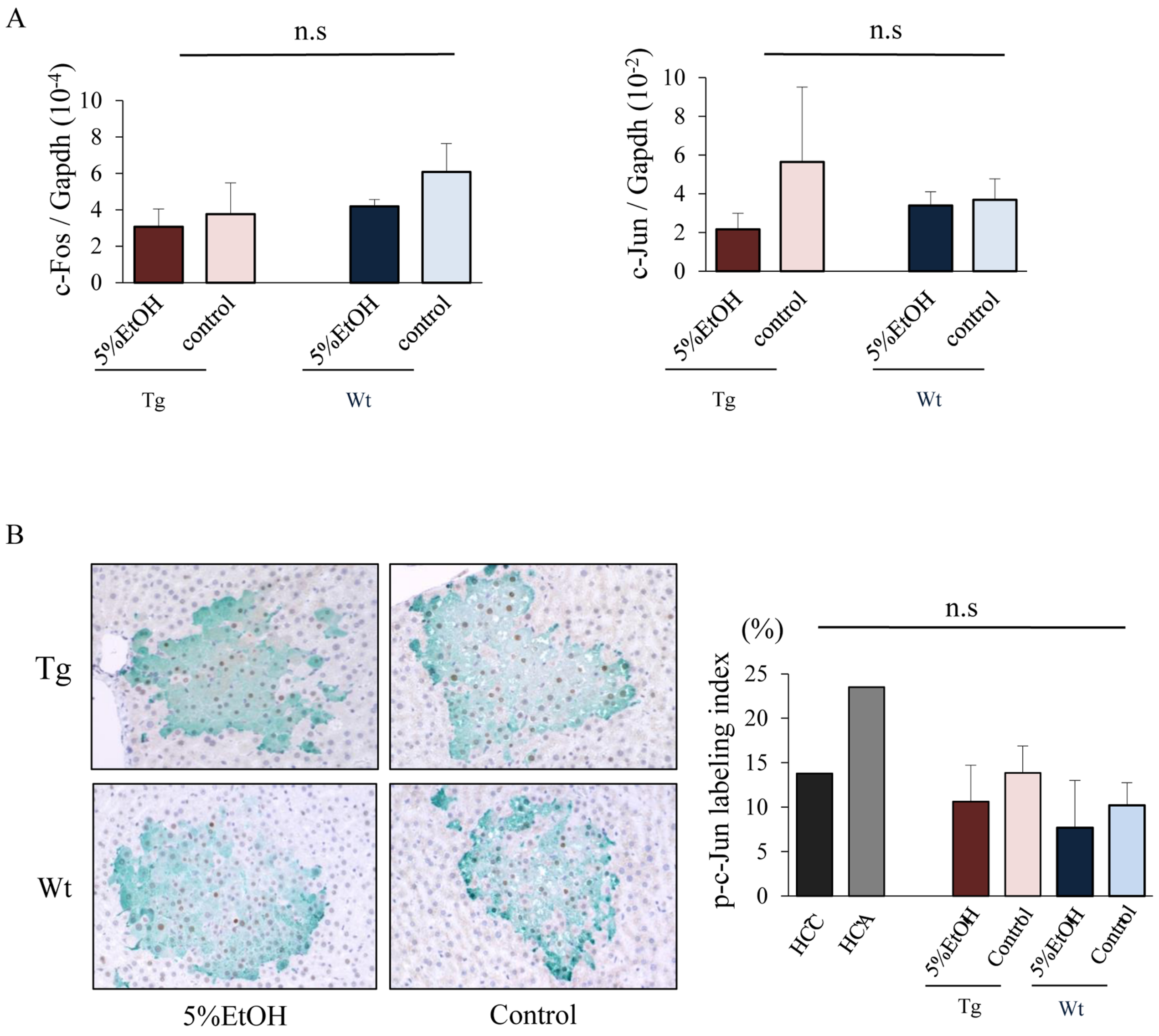

Supplementary Figure S3: AP-1 (c-Fos and c-Jun complex) expression was not altered by EtOH intake in both Tg and Wt rats. A. mRNA expression levels of c-Fos and c-Jun detected by quantitative RT-PCR. **B.** Immunohistochemical staining for p-c-Jun (brown) and GST-P (green). The percentage of p-c-Jun positive hepatocytes in HCC, HCA and GST-P positive foci in each group. Data are presented as mean ± SD, n = 4–5 per group.

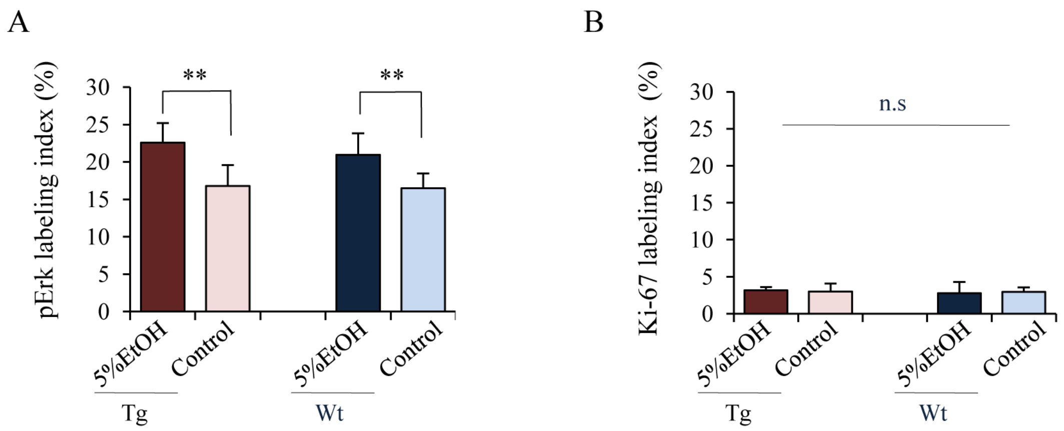

Supplementary Figure S4: EtOH enhanced pErk expression but did not altered Ki-67 labeling index in sinusoidal lining cells. Percentage of pErk **A.** and Ki-67 **B.** in sinusoidal lining cells. Date are presented as mean ± SD, $n = 5$ per group, at least 1,000 sinusoidal lining cells per rats. **: $P < 0.01$

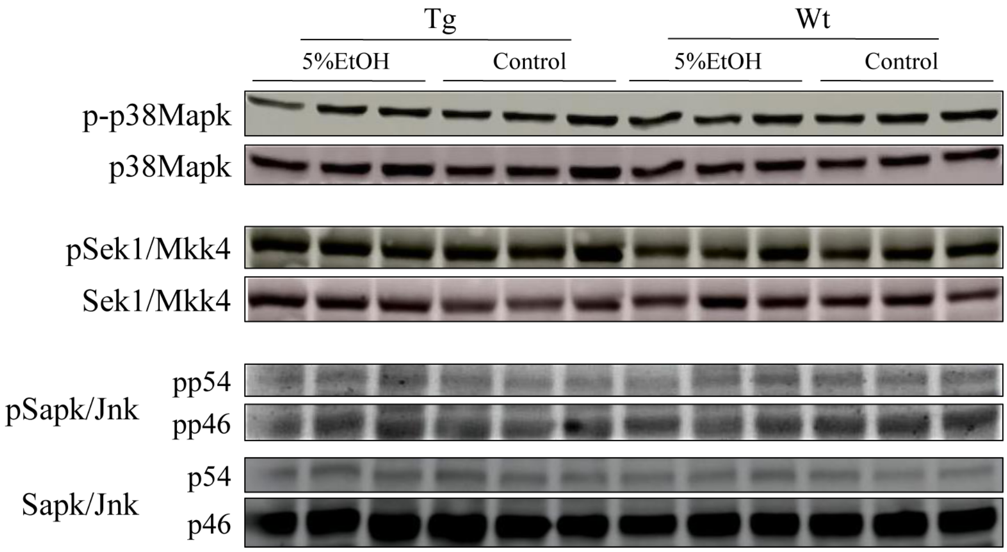

Supplementary Figure S5: The protein expression level of various components of the MAPK signaling pathway in Tg and Wt rats. Western blotting for p-p38Mapk (Thr180/Tyr182), p38Mapk, pSek1/Mkk4 (Ser80), Sek1/Mkk4, pSapk/Jnk (Thr183/Tyr185) and Sapk/Jnk.

Supplementary Table S1: Serum levels of hepatic enzymes, lipids and albumin in Tg and Wt rats.

		No.	Alb(g/dl)	ALP(U/L)	AST(U/L)	ALT(U/L)	γGTP(U/L)	LDH(U/L)	T-chol(mg/dl)
Tg	5%EtOH	12	4.1 ± 0.2	481 ± 108	63.3 ± 5.6	42.5 ± 7.5	1.6 ± 1.2	315 ± 183	60.1 ± 7.8
	1%EtOH	11	4.1 ± 0.1	498 ± 151	63. 7 ± 6.3	45.7 ± 6.1	1.7 ± 0.5	320 ± 176	69.4 ± 8.1
	Control	12	4.0 ± 0.1	510 ± 116	70.5 ± 10.4	54.1 ± 13.2	1.2 ± 0.1	307 ± 120	65.3 ± 6.7
Wt	5%EtOH	12	4.1 ± 0.1	635 ± 560	67.1 ± 29.7	61.9 ± 83.1	1.5 ± 1.5	247 ± 152	65.6 ± 13.1
	1%EtOH	12	4.1 ± 0.1	491 ± 148	67.9 ± 12.4	48.4 ± 11.0	0.8 ± 0.8	272 ± 134	67.8 ± 9.0
	Control	12	4.1 ± 0.1	541 ± 204	66.5 ± 8.1	44.8 ± 11.0	0.8 ± 0.4	374 ± 165	66.4 ± 12.7

Tg, connexin32 dominant negative transgenic; Wt, wild-type
Alb, Plasma albumin; ALP, alkaline phosphatase; AST, aspartate aminotransferase; ALT, alanine aminotransferase; γGTP, gamma-glutamyl transpeptidase; LDH, lactate dehydrogenase; T-chol, total cholesterol
